# Incidence of Non-Traumatic Subconjunctival Hemorrhage in a Nationwide Study in Taiwan from 2000 to 2011

**DOI:** 10.1371/journal.pone.0132762

**Published:** 2015-07-16

**Authors:** Dan-Ning Hu, Chih-Hsin Mou, Shih-Chun Chao, Ching-Yang Lin, Chan-Wei Nien, Pei-Tzu Kuan, Jost B. Jonas, Fung-Chang Sung

**Affiliations:** 1 Department of Ophthalmology, New York Eye and Ear Infirmary of Mount Sinai, Icahn School of Medicine at Mount Sinai, New York, NY, United States of America; 2 Department of Ophthalmology, Show Chwan Memorial Hospital, Changhua, Taiwan; 3 Institue of Clinical and Medical Science, China Medical University, Taichung, Taiwan; 4 Management Office for Health Data, China Medical University and Hospital, Taichung, Taiwan; 5 Institute of Electrical and Computer Engineering, National Chiao Tung University, Hsinchu, Taiwan; 6 Department of Ophthalmology, Medical Faculty Mannheim of the Ruprecht-Karls-University, Heidelberg, Germany; Bascom Palmer Eye Institute, University of Miami School of Medicine;, UNITED STATES

## Abstract

**Objective:**

To determine the nationwide incidence of non-traumatic subconjunctival hemorrhage (NTSCH) in Taiwan from 2000-2011 and to analyze the risk factors for NTSCH using a case-control analysis.

**Methods:**

This is a population-based cohort administrative database study. Randomly selected 1,000,000 residents from the Taiwan National Health Insurance Research Database in 2000 and followed for 12 years to determine the population incidence of NTSCH. Individuals with the first diagnosis of subconjunctival hemorrhage were identified by the corresponding International Classification of Diseases code (ICD) 372.72. Traumatic subconjunctival hemorrhages (ICD-9 codes 921, 871, 850-854 and 959.01) were excluded. The association of NTSCH with various demographic factors, comorbidities and use of medications was studied by a population based case-control analysis using data of 2008-2011.

**Results:**

A total of 67,720 patients with a first-time diagnosis of NTSCH were identified during the 12 years period. The mean annual incidence was 65 per 10,000 individuals (crude incidence) and 60 per 10,000 individuals (age- and sex-standardized incidence). The incidence rate of NTSCH was higher in women than in men, [men-women ratio: 0.80 (95% confidence interval: 0.78-0.81)]. The age-specific incidence decreased from childhood to the group of teenagers, after which it increased to a maximal value of 136.2 per 10,000 people in the age group of 60-69 years. Case-control analysis showed that comorbidities of hypertension, purpura and thrombocytopenia, and the use of aspirin were significantly associated with the risk of NTSCH.

**Conclusions:**

This study indicates that NTSCH is a common eye disease that occurs once in 167 individuals in a general East Asian population per year. It occurs more often in women than in men and the age-specific incidence peaked in the age group of 60-69 years. Hypertension, purpura and thrombocytopenia, and the use of aspirin are the major risk factors for NTSCH.

## Introduction

Subconjunctival hemorrhage is a common eye disease that is caused by the rupture of a conjunctival vessel, resulting in a local extravasation of blood into the subconjunctival tissue and subconjunctival episcleral space.[1–5] The condition usually becomes apparent in the externally exposed part of the bulbar conjunctiva, where the blood readily finds space in the loose subconjunctival tissue.[1–5] A subconjunctival hemorrhage is usually flat with sharply defined edges. The initially red hemorrhage turns orange and yellow when blood degradation and absorption take place, with absorption usually being complete at four to seven days after the hemorrhage.[5] Due to the benign natural course of the disorder, therapy is normally not necessary; however, a subconjunctival hemorrhage frequently causes considerable alarm to the patient, therefore, most affected patients may have sought medical help.[1–5]

Causes of subconjunctival hemorrhage are numerous, with local trauma being one of the most common etiologies. Most subconjunctival hemorrhage cases result from spontaneous rupture of a conjunctival vessel and is called non-traumatic subconjunctival hemorrhage (NTSCH) or spontaneous subconjunctival hemorrhage and could be caused by various factors or without obvious causes.[1–22] A small proportion of NTSCH are associated with systemic hemorrhagic diseases, including platelet and coagulation disorders;[1,2,5,7–9,16] anticoagulant or antiplatelet therapy; [2,4,5,10–16] systemic vascular disease such as arterial hypertension or diabetes mellitus [1–6,16] or other relevant disorders.[1,5,16–22]

Although the NTSCH events are relatively common, the prevalence and incidence in a general population have not been examined yet. We therefore explored the Taiwanese National Health Insurance (NHI) service database and searched for data on the incidence of NTSCH. The Taiwanese NHI was launched in 1995 and has been offering comprehensive medical care coverage to all residents of Taiwan. It includes health prevention, clinical care, hospitalization, resident care and social rehabilitation. The NHI covered 96.1% of the Taiwanese population in the year 2000, and the coverage rate has steadily increased to the end of 2010. The database of the NHI, the NHI Research Database, which has been collecting all registration files and claim data from all ambulatory patients and in-hospital patients, has been used by previous studies to analyze the incidence of various diseases such as central retinal artery occlusion.[23] Using the NHI Research Database, the purpose of our present study was to determine the incidence of NTSCH in the Taiwan population in the study period from January 2000 to December 2011, and to determine various factors that may have association with the occurrence of NTSCH.

## Methods

We obtained the NHI Research Database for research purposes after the identifications of patients and care providers had been converted into surrogate numbers. Institutional review board approval was waived for this study.[23] This study was conducted in adherence to the tenets of the Declaration of Helsinki. Out of the NHI beneficiaries from the year 2000, we randomly selected the data of 1,000,000 individuals. The participants in the studied group did not differ significantly in age or sex from the total group of NHI participants.

The population-based data for our study were obtained by using the annual outpatient claims and hospitalization discharge claims for the years 2000–2011. Cases with subconjunctival hemorrhage were identified according to the corresponding code 372.72 of the International Classification of Diseases (Ninth Revision, Clinical Modification, ICD-9-CM). Individuals with both subconjunctival hemorrhage and any reported trauma to the eye or brain which occurred within seven days of the subconjunctival hemorrhage (ICD-9 code 921, 871, 850–854 and 959.01) were excluded.

Incidence rates were calculated by dividing the number of new cases by the population covered.[24] Repeat episodes were not counted in this study. Averaged age-specific and sex-specific incidence rates were determined by dividing the new number of cases in each age and sex group by the age-specific and sex-specific population, followed by averaging these data from 2000 to 2011. The annual men-to-women crude incidence rates ratio and the 95% confidence interval (CI) were assessed using Poisson regression analysis.[25] Age-standardized and sex-standardized incidence was also counted annually using the world population in 2010 as the reference population (http://www.census.gov/population/international/data/worldpop/tool_population.php). The data analysis further calculated the overall age-specific incidence by age for the entire study period. Poisson regression analysis was used to calculate the incidence rate ratio and 95% CI for each age group relative to those aged less than 10 years.[25] We further performed a case-control study by selecting NTSCH patients newly diagnosed from 2008–2011, compared to 4-fold randomly selected individuals without the diagnosis of NTSCH. Univariate and multivariate logistic regression analyses were performed to measure the odds ratio (OR) of NTSCH associated with demographic status (age and sex), comorbidities (hypertension, diabetes mellitus, coagulation factor deficiency, purpura and thrombocytopenia) and use of medications (aspirin, clopidogrel and warfarin). ICD-9-CM codes for these comorbidities and drug code for medicine identification were presented in (S1 Table). A two-side *P*-value <0.05 was considered to be statistically significant. All statistical analysis were carried out using SAS version 9.1 (SAS Institute Inc., Cary, NC) and Microsoft Excel 2007 edition (Microsoft Office).

## Results

**Fig 1:**
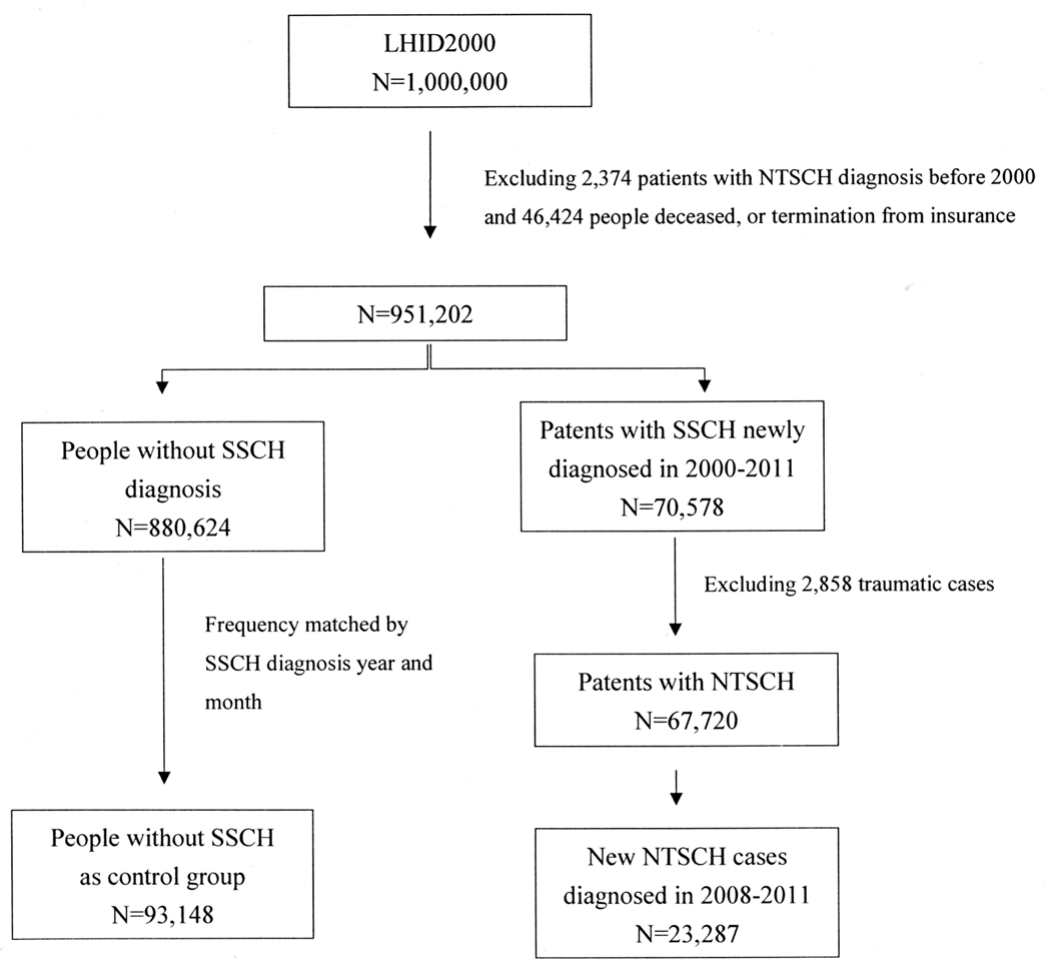
Flow Chart of Sample Selection for Case-control Study. SCH, Subconjunctival hemorrhage; NTSCH, Non-traumatic subconjunctival hemorrhage.

Out of the 1,000,000 eligible individuals, 951,202 (95.1%) participants were enrolled in our study after excluding 46,424 persons with incorrect demographic status, or termination from the insurance, or deceased in 2000; patients with NTSCH diagnosis before 2000 were also excluded (2,374 cases). During the 12-year study period from January 2000 to December 2011, a total of 70,578 new cases of NTSCH were identified. After excluding of 2,858 traumatic cases (2,054 cases of ocular injury and 804 cases of brain injury), there were 67,720 cases of NTSCH, including 37,241 women and 30,479 men (Fig 1 and Table 1).

**Table 1:** Annual Incident Cases and Crude Incidence Rates by Sex and Men-to-Women Rate Ratio for Non-Traumatic Subconjunctival Hemorrhage in Taiwan from 2000 to 2011.

Year	Total Population, x 10^4^			New Cases			Incidence Rate, per 10^4^			Men-to-Women Rate Ratio
	Women	Men	Total	Women	Men	Total	Women	Men	Total	(95% CI)
2000	46.59	48.53	95.12	2782	2391	5173	59.71	49.27	54.38	0.83 (0.78–0.87)
2001	45.82	47.51	93.34	2760	2446	5206	60.23	51.48	55.78	0.85 (0.81–0.90)
2002	45.00	46.55	91.55	3008	2579	5587	66.84	55.40	61.02	0.83 (0.79–0.87)
2003	44.22	45.67	89.88	2830	2574	54.04	64.00	56.37	60.12	0.88 (0.83–0.93)
2004	43.50	44.87	88.37	3255	2654	5909	74.83	59.15	66.87	0.79 (0.75–0.83)
2005	42.92	44.20	87.12	3156	2674	58.30	73.54	60.50	66.92	0.82 (0.78–0.87)
2006	42.36	43.54	85.90	313.5	2464	5599	74.02	56.59	65.18	0.76 (0.73–0.81)
2007	41.80	42.89	84.69	3248	2477	5725	77.70	57.76	67.60	0.74 (0.71–0.78)
2008	41.24	42.23	83.47	3172	2517	5689	76.92	59.60	68.16	0.77 (0.74–0.82)
2009	40.60	41.48	82.08	3090	2460	5550	76.12	59.30	67.62	0.78 (0.74–0.82)
2010	40.01	40.81	80.83	3444	2674	6118	86.07	65.52	75.69	0.76 (0.72–0.80)
2011	39.35	40.04	79.39	3361	2569	5930	85.41	64.16	74.69	0.75 (0.72–0.79)
Total	513.41	528.33	1041.73	37241	30479	67720	72.54	57.69	65.01	0.80 (0.78–0.81)

CI: confidence interval

The crude average incidence rate of NTSCH was 65.0 per 10,000 people per year. The age- and sex-standardized incidence rate of this disorder was 60.3 per 10,000 people per year (Table 2). The incidence rates did not differ significantly among 12 years included into the study period (*P* = 0.85, chi-square trend test).

**Table 2:** Age-Standardized Annual Incidence of Non-traumatic Subconjunctival Hemorrhage by Sex in Taiwan from 2000 to 2011.

	Incidence per 10^4^ Individuals*		
Year	Women	Men	Total
2000	60.08	49.85	54.91
2001	59.31	51.49	55.42
2002	64.86	55.15	60.00
2003	60.53	55.00	57.80
2004	70.77	56.62	63.65
2005	69.68	57.98	53.77
2006	62.62	49.56	56.07
2007	64.37	50.18	57.27
2008	62.44	50.51	56.47
2009	60.51	49.17	54.87
2010	67.01	55.13	61.12
2011	63.34	48.74	56.04
Total	65.79	54.76	60.28

*Standardized against the World Population in 2010 (http://www.census.gov/population/international/data/worldpop/tool_population.php).

The incidence rate of NTSCH was significantly (*P*<0.001) higher in women than in men. The men-to-women ratios varied from 0.74 (95% CI: 0.71–0.78) in 2007 to 0.88 (95% CI: 0.83–0.93) in 2003, with an average of 0.80 (95% CI: 0.78–0.81). The difference in incidence rates between women and men was statistically significant (*P*<0.001) for each year during the 12-year study period (Table 1).

The mean age-specific incidence rate of NTSCH was 35.6 per 10,000 people in the age group of <10 years of life, and decreased to 25.5 per 10,000 people in the age group of 10–19 years, which age group showed the lowest incidence as compared to any other age group. The incidence then increased and reached its peak in the 60–69 age group (136.2 per 10,000 populations). The difference in incidence rate of NTSCH among the different age-groups was statistically significant (*P*<0.001) (Table 3). Incidence rates were higher in females than in males for most of age groups, except children.

**Table 3:** Overall Sex- and Age-Specific Incidence of Non-Traumatic Subconjunctival Hemorrhages and Poisson Regression-Measured Rate Ratios in Taiwan.

Age Groups (Years)	New Cases	Incidence Rate per 10^4^	Ratio (95% Confidence Interval)
Overall			
< 10	2711	35.45	Reference
10–19	4048	25.45	0.72 (0.68–0.75)
20–29	5315	28.72	0.81 (0.77–0.85)
30–39	8257	45.30	1.28 (1.22–1.33)
40–49	14698	84.27	2.38 (2.28–2.48)
50–59	14899	121.84	3.44 (3.30–3.58)
60–69	9727	136.23	3.84 (3.68–4.01)
70–79	6282	126.84	3.58 (3.42–3.74)
≥ 80	1783	83.96	2.37 (2.23–2.51)
Female			
< 10	1194	32.56	Reference
10–19	1914	25.02	0.77 (0.71–0.83)
20–29	3036	33.47	1.03 (0.96–1.10)
30–39	4611	51.13	1.57 (1.47–1.67)
40–49	8293	95.42	2.93 (2.76–3.11)
50–59	8560	139.78	4.29 (4.04–4.56)
60–69	5429	148.54	4.56 (4.29–4.86)
70–79	3302	138.00	4.24 (3.97–4.53)
≥ 80	902	84.11	2.58 (2.37–2.82)
Male			
< 10	1517	38.12	Reference
10–19	2134	25.84	0.68 (0.63–0.72)
20–29	2279	24.16	0.63 (0.59–0.68)
30–39	3646	39.59	1.04 (0.98–1.10)
40–49	6405	73.20	1.92 (1.82–2.03)
50–59	6339	103.84	2.72 (2.58–2.88)
60–69	4298	123.31	3.23 (3.05–2.43)
70–79	2980	116.40	3.05 (2.87–3.25)
≥ 80	881	83.80	2.20 (2.02–2.39)

*P*-values were <0.001 by Poisson analysis.

For the case-control study, we identified 23,287 cases with NTSCH newly diagnosed in 2008–2011, including 13,064 women and 10,223 men. Controls were 93,148 persons randomly selected from general population without subconjunctival hemorrhage, frequency matched by diagnosis year and month of the cases.

The results of case-control analysis in Table 4 shows an adjusted OR of 1.38 (95% CI 1.33–1.41) for females, as compared with males with NTSCH. Compared with population aged <20 years, the risk of NTSCH peaked for the population with an age of 50–64 years with OR of 4.09 (95% CI 3.84–4.36). Hypertension, and purpura and thrombocytopenia were significantly associated with NTSCH, with adjusted ORs of 1.36 (95% CI 1.30–1.42) and 1.34 (95% CI 1.19–1.50), respectively. Coagulation factor deficiency was mildly associated with NTSCH, with crude and adjusted ODs at 1.53 (95% CI 1.26–1.85) and 1.20 (95% CI 0.98–1.46), respectively. Incidence of diabetes mellitus was significantly higher in the NTSCH group than in the controls, with a crude OR of 1.55 (95% CI 1.48–1.63); however, after adjusting for age, sex, other comorbidities and use of medications, the association was no longer statistically significant [adjusted OR was 0.98 (95% CI 0.94–1.04)]. Among various medications, NTSCH was significantly associated with the use of aspirin, with an adjusted OR at 1.09 (95% CI 1.05–1.13). Both the use of clopidogrel and warfarin were significantly associated with NTSCH, but the association was only marginal significant after the adjustment (Table 4).

**Table 4:** Case-control Analysis for Non-Traumatic Subconjunctival Hemorrhages Associated with Comorbidity and Medication History.

	NTSCH		Odds Ratio	
	No (N = 93148)	Yes (N = 23287)	(95% CI)	
	%	%	Crude	Adjusted*
Sex				
Female	48.0	56.1	1.38 (1.34–1.42)	1.38 (1.34–1.42)
Male	52.0	43.9	Ref.	Ref.
Age, year				
< 20	14.9	6.08	Ref.	Ref.
20–49	51.8	40.8	1.93 (1.82–2.05)	1.91 (1.80–2.03)
50–64	18.0	33.6	4.57 (4.30–4.86)	4.09 (3.84–4.36)
> = 65	15.3	19.6	3.13 (2.94–3.34)	2.46 (2.29–2.64)
Mean (SD)	42.7 (20.6)	50.3 (16.9)		
Comorbidity				
Hypertension	18.6	30.5	1.92 (1.86–1.98)	1.36 (1.30–1.42)
DM	7.96	11.8	1.55 (1.48–1.63)	0.98 (0.94–1.04)
CFD	0.41	0.62	1.53 (1.26–1.85)	1.20 (0.98–1.46)
PT	1.14	1.83	1.62 (1.45–1.82)	1.34 (1.19–1.50)
Medication history				
Aspirin	25.9	31.0	1.28 (1.24–1.32)	1.09 (1.05–1.13)
Clopidorgrel	1.83	2.90	1.61 (1.47–1.76)	1.05 (0.95–1.16)
Warfarin	0.94	1.53	1.65 (1.46–1.87)	1.10 (0.97–1.26)

* Adjusted for age, sex, comorbidity and medication history

DM, diabetes mellitus; CFD, coagulation factor deficiency; PT, purpura and thrombocytopenia; Aspirin included aspirin and acetylsalicylic acid

## Discussion

Case ascertainment in the present study was based on ICD-9-CM coding. Code 372.72 is conjunctival hemorrhage. Most conjunctival hemorrhage cases are subconjunctival hemorrhage; bleeding from the conjunctiva is very rare and often associated with trauma.[1,5,16] Therefore, after the exclusion of traumatic cases, the patients identified in this study were virtually those with subconjunctival hemorrhage. In this large population-based study, the mean incidence of reported NTSCH was 65.0 per 10,000 people per year, with an increase from 25.5 per 10,000 people in the age group of 10–19 years to 136.2 per 10,000 people in the age group of 60–69 years. The incidence of reported hemorrhages was stable during the 12-year study period.

Since incidence rates of NTSCH have not been reported from other countries yet, our data cannot directly be compared with the findings obtained in other similar investigations. Our study found that the incidence of NTSCH was significantly higher in women than in men with a men-to-women ratio of 0.80 (95% CI: 0.78–81). The higher incidence rate of this disorder in women may be related to several causes of subconjunctival hemorrhage that are only present in women, such as postpartum conditions; or which are more prevalent in women, such as idiopathic thrombocytopenic purpura.[1,5,6,7,16] In our case-control study, the incidence of thrombocytopenic purpura in the female was higher than that in the male; however, this difference was not statistically significant (data not shown). Therefore, the causes of difference of incidence of NTSCH between the male and female requires further investigation. The difference of health care use patterns of people between the female and male (if exist) may also play a role in this difference.

The incidence of NTSCH was lowest in the age group of 10–19 years (25.2 per 10,000 individuals). The relatively high incidence of NTSCH in the age group of 0–9 years (35.5 per 10,000) may have been due to the relatively high occurrence of NTSCH of 10%-30% in newborns.[20–22] At an age of 20+ years, the incidence of NTSCH gradually increased, parallel to an increase in the occurrence of various disorders which can cause NTSCH, such as thrombocytopenia, hypertension and administration of various anticoagulants and anti-platelet drugs [1–15,16]. The incidence reached a peak of 1.36 new cases in 100 subjects in the age group of 60–69 years.

The results of case-control analysis in Table 4 shows an adjusted OR of 1.38 (95% CI 1.33–1.42) for females, compared with males for the disease. [1–6,16] Compared with population < 20 years, the risk of NTSCH peaked for population of 50–64 years old with an adjusted OR of 4.09 (95% CI 3.84–4.36). It has been reported that hypertension was common in NTSCH. Fukuyama et al. reported that in 225 subconjunctival hemorrhage patients, 36 cases had hypertension.[1] They listed hypertension as one of the most common causes of subconjunctival hemorrhage.[1] However, they did not compare the prevalence of hypertension in subconjunctival hemorrhage group with a control group, therefore, it is difficult to evaluate the role of hypertension in the occurrence of subconjunctival hemorrhage. In the present study, hypertension was significantly associated with NTSCH. The fragibility of conjunctival vessels in hypertension may cause rupture of vessels and bleeding into the subconjunctival space. Diabetes is also a common associated condition of NTSCH, [1,4,5,16] however, it is not a significant risk factor of NTSCH based on the present study. Purpura (including thrombocytopenia and non-thrombocytopenia) and coagulation factor defects (including hemophilia and other coagulation factors deficiency) have been reported as the causes of NTSCH.[1,2,5,7–9,16] However, no population based case-control study has been performed to verify this association. In the present study, purpura and thrombocytopenia were significantly associated with NTSCH, with an adjusted OD at 1.34 (95% CI 1.19–1.50). Part of NTSCH cases (0.62%) were associated with coagulation factor defects, which was significantly higher than that in the controls (0.41%). However, after adjusted for various factors, this association was only marginal, with an adjusted OR at 1.20 (95% CI 0.98–1.46).

Use of aspirin, clopidogrel or warfarin has been mentioned as the risk factors for the occurrence of NTSCH. [4,5,10–15] However, no population based case-control study has been reported to verify this association. Use of aspirin was significantly associated with NTSCH in the present case-control study, with an adjusted OR at 1.09 (95% CI 1.05–1.13); whereas the use of clopidogrel or warfarin was only marginal associated with NTSCH (Table 4).

The present study found that the occurrence of NTSCH was associated with several systemic diseases and the use of relevant medications. It was reported that NTSCH could be the first clinical feature of a severe systemic disease.[5,7] Therefore, in individuals with NTSCH, especially the patients with persisting or recurrent NTSCH, a careful medical evaluation for the presence of unnoticed arterial hypertension and hemorrhagic diseases is warranted.[1,5,7] The appearance of a NTSCH in patients taking aspirin or anticoagulant medications require a consultation with their physicians to consider if this medication should be discontinued or the dosage should be adjusted.[5,12,13]

Potential limitations of our study should be mentioned. First, the database included only the occurrence of reported hemorrhages while those bleedings that were not diagnosed or not reported still remained unnoticed. However, a NTSCH frequently causes considerable alarm to the patients, most patients may have sought medical help. In addition, the NHI in Taiwan provides an opportunity for all patients to see an ophthalmologist immediately without a waiting period. Second, the case ascertainment was based on the coding in the International Classification of Diseases and any hemorrhage coded in another manner, was not taken into account in our study. Third, as in any study, the results of the statistical analysis depend on the robustness of the primary data, *i*.*e*., in our study on the accuracy with which the primary medical doctors coded diseases such as NTSCH. Since the clinical appearance of a NTSCH is clear and characteristic, the diagnosis might have been straight forward for most doctors. Fourth, the present study was conducted in a specific population, further studies in other countries or districts are required to see if this incidence rate could be referenced for other different populations with different ancestries.

Strengths of this study include the use of a nationwide database with a large sample size and almost complete coverage of the population, and the assessment of a well-defined disease.

In conclusion, a NTSCH occurs in one out of 167 individuals in a general East Asian population per year. It occurs more often in women than in men. Its incidence decreases from childhood to the group of teenagers, after which it increases to a maximal value of 136 per 10,000 people in the age group of 60–69 years. Hypertension, purpura, thrombocytopenia and the use of aspirin are significantly associated with the risk of NTSCH.

## Supporting Information

S1 Table ICD-9-CM Code for Disease and Drug Code for Medicine Identification. (DOC)
