# Supplementary material for: Incidence of Non-Traumatic Subconjunctival Hemorrhage in a Nationwide Study in Taiwan from 2000 to 2011
Source: PLoS One. 2015 Jul 16;10(7):e0132762. doi: 10.1371/journal.pone.0132762 (PMC4504497; doi:10.1371/journal.pone.0132762)
Supplement: S1 Table — (DOC) [file pone.0132762.s001.doc]

**Supporting Table**

**Table 1. ICD-9-CM Code for Disease and Drug Code for Medicine Identification**

| Disease | ICD-9-CM code |
| --- | --- |
| Hypertension | 401-405 |
| DM | 250 |
| CFD | 286 |
| PT | 287 |
| Medicine | Drug code |
| Aspirin | A002107500, A042461100, AC43309100, A030784100, A0485421G0, A003092100, A0424611G0, AC433091G0, A032045100, A0495361G0, A004813100, A042774100, AC43663100, A033292100, A054863100, A006485500, A042915100, AC436631G0, A034028500, A0548631G0, A013465500, A0429151G0, AC43664100, A034975100, A0551041G0, A015219500, A042934100, AC436641G0, A036599100, AC37344100, A019702500, A0429341G0, AC44176100, A037344100, AC373441G0, A021870100, A043139100, AC441761G0, A0373441G0, AC37702100, A023330100, A043142100, AC48310100, A037702100, AC41220100, A023534100, A043212100, AC48542100, A040658100, AC41511100, A024465100, A043254100, AC495361G0, A040665100, AC415111G0, A025160100, A043309100, AC548631G0, A0406651G0, AC41814100, A025848100, A0433091G0, AC54985100, A040826100, AC418141G0, A025855500, A043663100, AC549851G0, A041018100, AC42461100, A026164100, A0436631G0, B008262100, A041073100, AC424611G0, A026523500, A043664100, B017332100, A041220100, AC42774100, A026523563, A0436641G0, B018755100, A041511100, AC43139100, A027304500, A044016100, B020365100, A0415111G0, AC43142100, A028108100, A0440161G0, B023619100, A041814100, AC43212100, A028591100, A044069100, B023919100, A0418141G0, AC43254100, A028936100, A044176100, B024025100, A029219500, A0441761G0, B0240251G0, A029536100, A044578100, B025326100, A029740100, A045015100, BC24025100, A029754100, A045072100, BC240251G0, A029788100, A048310100, BC25326100, A029888100, A048339100, C001621100, A030696100, A0483391G0, N004155100, A030775100, A048542100, N011693100 |
| Clopidorgrel | A047589100, A048062100, A048649100, A048730100, A049224100, A049344100, A049719100, A049967100, A050126100, A050241100, A052522100, A055026100, A055044100, A055428100, A057123100, A057140100, AA48730100, AA57140100, AB48649100, AB48730100, AB49344100, AB49719100, AB50126100, AB57819100, AC48062100, AC48649100, AC49224100, AC49344100, AC49719100, AC49967100, AC50126100, AC50241100, AC52522100, AC55026100, AC55428100, AC57140100, AC57819100, AC58093100, B022932100, B024863100, B025034100, B025114100, B025222100, B025326100, B026190100, B026335100, BC22932100, BC24863100, BC25034100, BC25114100, BC25222100, BC25326100, BC25873100, BC26190100, BC26252100, BC26335100 |
| Warfarin | A043862100, A050095100, A050423100, A052559100, AC43862100, AC50095100, AC50423100, AC52559100, AC55271100, B020346100, B020354100, B020515100, B020516100, B023426100, B023572100, B023573100, BC23572100, BC23573100 |

DM, diabetes mellitus; CFD, coagulation factor deficiency; PT, purpura and thrombocytopenia
